# Polyelectrolyte–Surfactant Complex Nanofibrous Membranes for Antibacterial Applications

**DOI:** 10.3390/polym16030414

**Published:** 2024-02-01

**Authors:** Qiaohua Qiu, Zhengkai Wang, Liying Lan

**Affiliations:** College of Textile Science and Engineering, Zhejiang Sci-Tech University, Hangzhou 310018, China; wangzhengkaiii@163.com (Z.W.); lanying2024@163.com (L.L.)

**Keywords:** hydrolyzed polyacrylonitrile, quaternary ammonium salt, polyelectrolyte, surfactant, antibacterial

## Abstract

Polyelectrolyte–surfactant complexes (PESCs) have garnered significant attention due to their extensive range of biological and industrial applications. Most present applications are predominantly used in liquid or emulsion states, which limits their efficacy in solid material-based applications. Herein, pre-hydrolyzed polyacrylonitrile (HPAN) and quaternary ammonium salts (QAS) are employed to produce PESC electrospun membranes via electrospinning. The formation process of PESCs in a solution is observed. The results show that the degree of PAN hydrolysis and the varying alkyl chain lengths of surfactants affect the rate of PESC formation. Moreover, PESCs/PCL hybrid electrospun membranes are fabricated, and their antibacterial activities against both Gram-negative Escherichia coli (*E. coli*) and Gram-positive Staphylococcus aureus (*S. aureus*) are investigated. The resulting electrospun membranes exhibit high bactericidal efficacy, which enables them to serve as candidates for future biomedical and filtration applications.

## 1. Introduction

Complexes of oppositely charged polyelectrolytes and surfactants are interesting systems because the polyelectrolyte–surfactants complexes (PESCs) form various states such as liquids, gels, and solids by their molecular structure and composition [1,2]. The complexation process is driven primarily by intermolecular interactions, both electrostatic and hydrophobic [3,4]. These interactions depend on a variety of molecular parameters, including polyelectrolyte charge density, polyelectrolyte molecular weight, functional groups, and surfactant architecture [3,5]. Such complexes are of great significance due to their wide-ranging applications in industries such as personal care [6,7], food additives [8,9], and pharmaceutical products [10,11]. However, most applications of PESCs are primarily focused on liquid or emulsion states, limiting their utility in solid material-dependent applications such as filtration materials and wound dressings. Therefore, exploring the use of PESCs in solid form will extend the range of their potential applications.

Electrospinning has attracted a significant amount of interest because it is a simple process that enables the manufacture of highly porous mats composed of fibers with a high surface-to-volume ratio that can be easily tailored for specific applications [12,13,14,15]. Polyelectrolytes are a fascinating group of polymers that contain ionizable groups capable of dissociating into multi-ionic chains and free counter ions with opposing charges in polar solvents [16,17]. Due to their ionic properties, the electrospinning of polyelectrolyte fibers is a more challenging process compared to neutral polymers. At present, there exist several techniques for fabricating polyelectrolyte-based electrospun fibers. For instance, the addition of organic solvents such as dimethylamide (DMF) or methanol can neutralize the polyelectrolyte structure and facilitate fiber formation [18]. Another approach involves the formation of a polyelectrolyte complex (PEC) by mixing polymers with opposite charges, followed by plasticizing the PEC with salt and electrospinning the resulting liquid complex coacervates into fiber mats [19,20]. Alternatively, it may be blended with neutral polymers such as polyethylene oxide (PEO) to enhance spinnability [21]. To our knowledge, electrospun fibers based on polyelectrolyte –surfactant complexes have been less explored, particularly to provide them with antibacterial capabilities. 

The emergence of infectious diseases leading to public health outbreaks is currently a primary focus of global security concerns. Developing antibacterial materials that can effectively eliminate harmful microorganisms or impede their proliferation and reproduction holds great promise as a strategic approach for providing biological protection. The fabrication of antibacterial nanofibers typically involves incorporating biocides into the fiber matrix. The achievement of this can be facilitated by mixing the active agent within the polymer solution prior to electrospinning, confining the active agent exclusively to the core region of the fibers through coaxial electrospinning, or subjecting the fibers to post-treatment procedures subsequent to electrospinning. The utilization of various widely recognized active agents, such as antibiotics, triclosan, quaternary ammonium salts (QAS), biguanide, and metal oxide and particles, has been observed [22,23,24,25]. The QAS represent a significant class of organic antibacterial agents that possess the ability to effectively inhibit the growth of both Gram-negative and Gram-positive bacteria [26,27]. The apparent positive electrical properties of QAS facilitate its easy combination with cationic polyelectrolytes, thereby imparting antibacterial properties to the matrix.

**Scheme 1:**
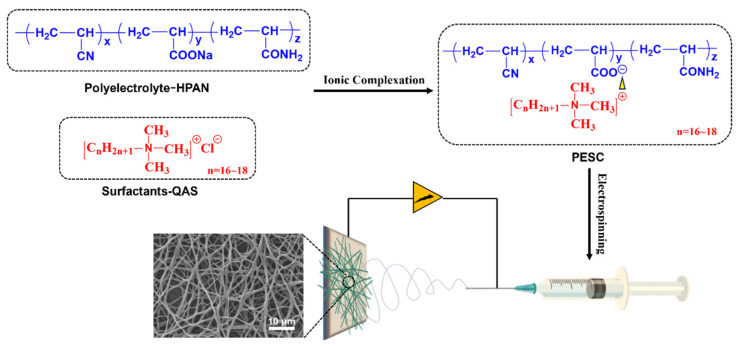
Schematic illustration of the synthesis of PESCs and the fabrication process of PESC electrospun fibers.

Here, we investigate electrospun nanofibers based on PESCs. A scheme for the electrospinning process is shown in Scheme 1. Hydrolyzed polyacrylonitrile (HPAN) was employed as the polyelectrolyte, while QAS, a cationic surfactant possessing antibacterial properties, served as the utilized surfactant. The investigation focused on the influence of concentration of NaOH solution, mass ratio of PAN-NaOH, addition sequence, and alkyl chain length on the precipitation behavior of PESCs. Additionally, PESCs and PESCs/PCL nanofibrous membranes were fabricated, and the antibacterial efficacy of PESCs/PCL against Gram-negative *Escherichia coli* (*E. coli*) and Gram-positive *Staphylococcus aureus* (*S. aureus*) was investigated. These PESC fiber mats provide insights into the design and construction of solid PESC with antibacterial functions.

## 2. Materials and Methods

### 2.1. Materials

PAN (Mw 85,000) was purchased from Shanghai Chemical Fibers Institute (Shanghai, China). Quaternary ammonium salts with different alkyl chain lengths, Dodecyltrimethylammonium chloride (DTAC, 98%), Tetradecyltrimethylammonium chloride (TTAC, 98%), Hexadecyltrimethylammonium chloride (CTAC, 98%), and Stearyl Trimethyl Ammoium Chloride (STAC, 98%), were purchased from Aladdin, China. *E. coli* (ATCC 8099) and *S. aureus* (ATCC 6538) were purchased from Nanjing Clinical Biological Technology Co., Ltd. (Nanjing, China). Other reagents and solvents were of analytical grade and used as received.

### 2.2. Preparation of PESC Polymers

The preparation of PESC antibacterial polymers was achieved according to our previously reported procedure [28]. In a typical synthesis, the appropriate amounts of PAN powder and NaOH solution were added into a three-necked flask equipped with a reflux condenser and mechanical mixture. Thereafter, the reaction temperature was increased to 100 °C and kept for 3 h. The pH of the above solution was adjusted to 6.5 with 1 M HCl, and the polyelectrolyte solution was finally obtained. The concentration of the HPAN solution was adjusted to 8 mg mL^−1^ using deionized water for further use.

Subsequently, aqueous solutions of quaternary ammonium salts with varying alkyl chain lengths (DTAC, TTAC, CTAC, and STAC) at a concentration of 0.03 M were prepared and titrated into the as-prepared polyelectrolyte solution until coagulation occurred. The resulting precipitates, known as the solid PESCs (HPAN-DTAC, HPAN-TTAC, HPAN-CTAC, and HPAN-STAC), were purified by filtering followed by multiple washes with DI water before being vacuum dried for further use.

### 2.3. Preparation of PESCs and PESCs/PCL Electrospun Fibers

The electrospinning solution of PESCs was prepared by dissolving solid PESCs (HPAN-DTAC, HPAN-TTAC, HPAN-CTAC, and HPAN-STAC) in a mixture of isopropanol and chloroform (weight ratio of 5:5) at room temperature with stirring for overnight; the concentration of PESCs was 10 wt %. The electrospinning solution of PESCs/PCL was prepared by dissolving solid PESCs and PCL (weight ratio of 5:5) in a mixture of isopropanol and chloroform (weight ratio of 5:5) at room temperature with stirring overnight; the concentration of PESCs/PCL was 10 wt %. Afterward, the solutions of PESCs and PESCs/PCL were transferred to a 5 mL syringe and fed at a rate of 0.8 mL h^−1^ by a programmable syringe pump, respectively. A high voltage of 8 kV was applied to the needle, resulting in the formation of a continuously charged polymer jet. The prepared PESCs and PESC/PCL nanofibrous membranes were deposited on aluminum foil at a 15 cm distance and then dried in a vacuum at 30 °C for 24 h to remove the residual solvent.

### 2.4. Analysis of the Formation Process of PESCs

To observe the precipitation behavior of PESCs, transmittance and conductivity were determined using an ultraviolet spectrophotometer (UV, PerkinElmer-Lambda 35) and conductivity meter at room temperature (Shanghai YUEPING, DDS-307), respectively. Briefly, the solution concentrations of HPAN and QAS were 8 mg mL^−1^ and 40 mg mL^−1^, respectively. At room temperature, a certain volume of HPAN aqueous solution was introduced into a beaker, followed by the gradual addition of QAS solution with stirring to ensure homogeneity. The contents of HPAN and QAS added are presented in Table S1 (in Supplementary Material).

### 2.5. Characterization

The structures of HPAN and HPAN-QAS were characterized using Fourier transform infrared spectroscopy (FTIR, Thermo Fisher Antaris II, Thermo Fisher Scientific, Waltham, MA, USA). Thermogravimetric analysis (TAG, TGA400, Perkin-Elmer, Shelton, CT, USA) was performed using a thermogravimetric analyzer at a heating rate of 15 °C min^−1^ ranging from room temperature to 500 °C under a N_2_ atmosphere. After being coated with platinum, the surface morphologies of PESCs and PESCs/PCL electrospun fibers were examined using a scanning electron microscope (FESEM, TM300, Hitachi, Tokyo, Japan). The diameter distributions of nanofibers were calculated using Image J software (Java 1.8.0_345, NIH, Bethesda, MD, USA). The mechanical properties were evaluated using a tensile property tester with a pretension of 0.2 cN and a constant tensile speed of 10 mm min^−1^. The samples, all measuring 50 mm × 5 mm, were clamped at a distance of 10 mm.

### 2.6. Antibacterial Activity Assessment

Typical bacterial microorganisms, including Gram-negative *E. coli* and Gram-positive *S. aureus*, were selected for the antibacterial activity assessment. The bacteria were cultivated in an oven at 37 °C overnight for activation. After bacterial activation, 12 mg of each sample was dispersed into a conical flask containing 5 mL of a phosphate-buffered saline (PBS) solution and 5 × 10^5^ CFU mL^−1^ of bacteria. The solutions were incubated on a shaking bed at 37 °C overnight. After that, 100 µL of the mixture was dispersed onto LB agar plates and incubated at 37 °C for 18 h. Photographs and the numbers of the bacteria colonies grown on agar plates were obtained to assess their antibacterial performance. 

The inhibition activity has been assessed by agar diffusion assay. The bacterial suspension (100 µL, concentration of ~10^5^ CFU mL^−1^) was spread on LB agar plates, and PESC/PCL nanofibrous membranes with a radius dimension of 1 cm were sterilized under UV light for 1 h. Then the nanofibrous membranes were placed on the surface of the agar. After incubating for 18 h at 37 °C, the zone of inhibition (ZOI) of *E. coli* and *S. aureus* was observed.

## 3. Results and Discussion

### 3.1. Effects of NaOH Solution Concentration and PAN-to-NaOH Mass Ratio on PESC System

The concentration of NaOH solution and the mass ratio of PAN-to-NaOH influenced the number of carboxyl groups (-COOH) on the hydrolyzed PAN chain, which determines the charge density of polyelectrolyte and subsequently affects its interaction with surfactants. CTAC was chosen as the surfactant and added dropwise to the HPAN solution. The combination process of PESC was characterized using a conductivity meter and ultraviolet spectrophotometer. PAN was hydrolyzed with 6%, 8%, and 10% NaOH solutions, respectively. The mass ratio of NaOH to HPAN was 1:1, with hydrolysis taking place at 100 °C for 3 h. After the initiation of PAN hydrolysis, the -CN groups were rapidly hydrolyzed to form -CONH_2_, which subsequently continued to hydrolyze into -COO-, resulting in the formation of a negatively charged polyelectrolyte [29,30]. 

However, due to steric hindrance from neighboring groups, both generated -COO- and -OH have identical charges that impede further hydrolysis towards adjacent -CN and -CONH_2_. The concentration increase in NaOH solution could overcome the adjacent group repulsion effect, enhance the hydrolysis degree of PAN, generate more -COO-, and elevate the conductivity of polyelectrolyte solution. 

**Figure 1:**
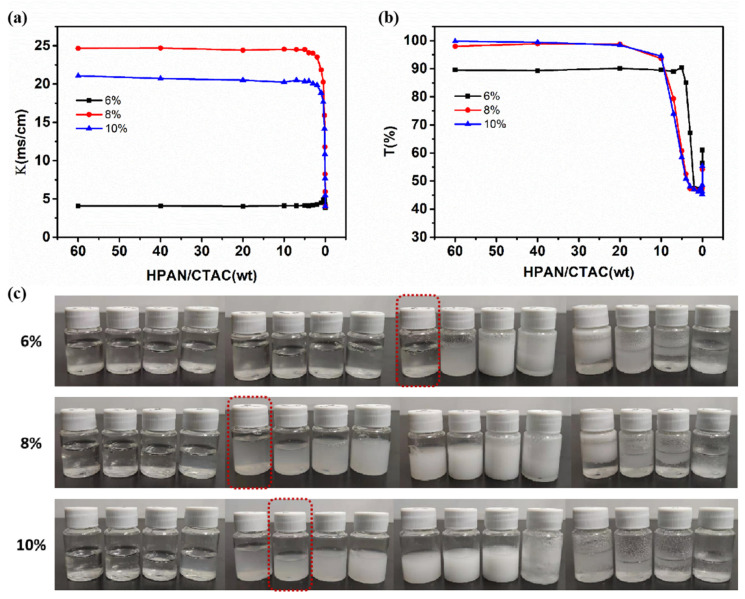
(**a**) Conductivity and (**b**) T% of HPAN-CTAC solution with different NaOH solution concentrations. (**c**) Photographs of the transformation of HPAN-CTAC solution (the point at which the solution system begins to exhibit turbidity is indicated by the dotted red box).

As shown in Figure 1a, when the concentration of NaOH solution was increased from 6% to 8%, the conductivity of the initial solution exhibited a significant increase from 4.07 ms/cm to 24.7 ms/cm. Notably, excessive concentration of the NaOH solution led to a decrease in the conductivity of the HPAN solution, which may be attributed to the macromolecular skeleton breakage of PAN caused by a high NaOH solution, resulting in -COO- loss. Moreover, an increase in the amount of NaOH was not conducive to uniform mixing with PAN, thereby affecting the complete hydrolysis reaction. 

Furthermore, the conductivity of the composite solution initially remained relatively stable. The reason is most likely that at this stage, the concentration of the surfactant is low, and polyelectrolyte exerts a dominant influence in the mixed solution. On the other hand, due to the initial concentration of CTAC (40 mg mL^−1^) being much higher than its CMC value (1.152 mg mL^−1^, Figure S1), it entered the HPAN polyelectrolyte solution in a micellar state, with hydrophobic cetyl groups being distributed within the core while positively charged head groups towards the shell. As a result, the micelles exhibited a relatively high surface charge density, which hindered the contact between the molecular chain of HPAN and more CTAC heads. In addition, the conductivity of CTAC in its micellar state exhibited a slow increase with the concentration, resulting in minimal changes to the total number of ions present within the system. In other words, the conductivity of the system remained largely unchanged. As the concentration of CTAC increased, the conductivity of the system experienced a sudden decrease. This phenomenon was attributed to the intensified electrostatic interaction between CTAC and HPAN, leading to complex formation and reduced ion concentration in solution, ultimately resulting in a sharp decline in conductivity.

In addition, the formation process of the HPAN-CTAC complex was investigated using the turbidity method. The light transmittance (T%) of the composite solution was measured at 500 nm using an ultraviolet spectrophotometer. Neither the HPAN nor CTAC exhibited any absorption at this wavelength. As shown in Figure 1b, the initial T% varies with different concentrations of NaOH. Inadequate hydrolysis of PAN occurred when the concentration of NaOH solution was 6%, resulting in some undissolved substance and a subsequent decrease in initial solution absorbance. The overall trend could be classified into three stages (Figure 1b,c). During the first stage, when HPAN/CTAB (wt) ratios were less than 5 (NaOH solution was at 6%), 20 (NaOH solution was at 8%), and 20 (NaOH solution was at 10%), respectively, the transmittal rate remained relatively stable, indicating a homogeneous dilute solution system. As the HPAN/CTAB (wt) ratio increased, there was a sharp decrease in transmittance. The minimum value was attained when the HPAN/CTAB (wt) ratios reached 0.5 (NaOH solution was at 6%), 1 (NaOH solution was at 8%), and 1 (NaOH solution was at 10%), respectively, leading to system condensation. The mixed solution system then transformed into a white emulsion, followed by layering where the upper part was supernatant and the lower part sediment. With an increase in the amount of CTAC surfactant solution, the hydrophobic interaction between surfactants strengthened, while the hydrophobicity within the HPAN-CTAC system weakened. This resulted in partial condensation dissolving and the solution becoming colorless and transparent again, with increased transmittance [31]. Since HPAN and CTAB are not absorbed at 500 nm, a decrease in transmittance indicates polyelectrolyte–surfactant complex formation. 

**Figure 2:**
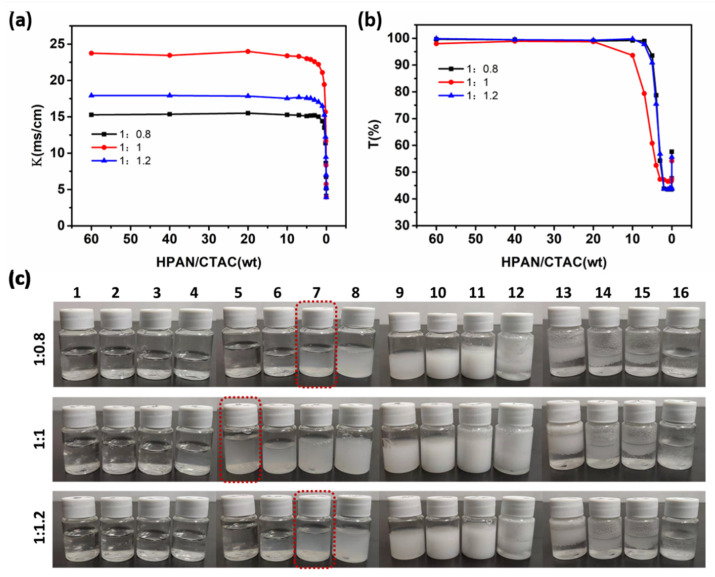
(**a**) Conductivity and (**b**) T% of HPAN-CTAC solution with different mass ratios of PAN to NaOH. (**c**) Photographs of the transformation of HPAN-CTAC solution (the point at which the solution system begins to exhibit turbidity is indicated by the dotted red box).

Similarly, the mass ratio of PAN-to-NaOH also had a great influence on the HPAN-CTAC composite system. The mass ratio of PAN to NaOH was 1:0.8, 1:1, and 1:1.2, respectively. All other conditions remained constant, including the concentration of NaOH solution at 8%, the hydrolysis time at 3 h, and the hydrolysis temperature at 105 °C. As depicted in Figure 2a, a decrease in the mass ratio of PAN and NaOH results in an increase in the mass percentage of NaOH, leading to a higher concentration of -OH within the solution system. This subsequently accelerated the hydrolysis rate of PAN, resulting in an increased generation of -COO- groups and enhanced conductivity within the HPAN polyelectrolyte solution. However, the high concentration of NaOH solution could lead to the break of the PAN molecular chain segment. For the transmittance of the HPAN-CTAC composite system (Figure 2b,c), a relatively complete hydrolysis reaction of PAN and an increased number of ions in the solution system were observed when the mass ratio of PAN to NaOH was 1. As a result, the probability of the positive quaternary ammonium charge of CTAC binding with the -COO- in HPAN increased, facilitating the rapid formation of the HPAN-CTAC complex. This led to an increase in turbidity and a decrease in solution system permeability. 

### 3.2. Effects of Raw Material Adding Sequence on the PESC System

**Figure 3:**
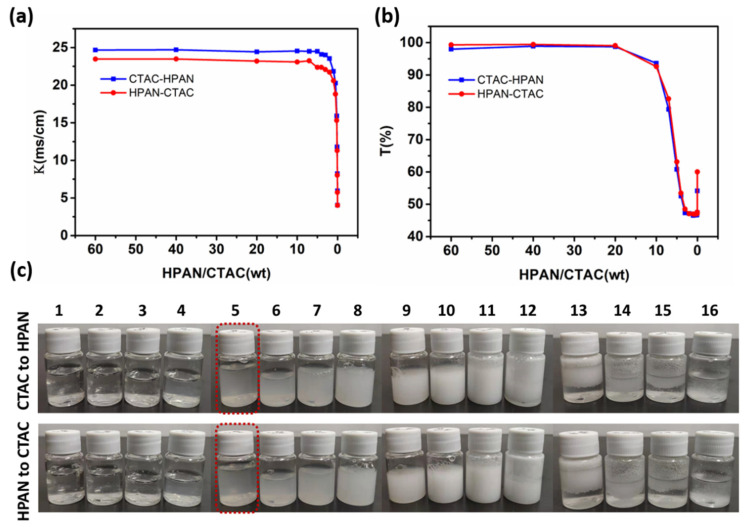
(**a**) Conductivity and (**b**) T% of HPAN-CTAC solution with different raw material addition order. (**c**) Photographs of the transformation of HPAN-CTAC solution (the point at which the solution system begins to exhibit turbidity is indicated by the dotted red box).

To investigate the impact of raw material addition orders on HPAN-CTAC complex formation, we examined the evolution of the HPAN-CTAC composite system when CTAC solution was added to HPAN electrolyte solution and vice versa. All other conditions remained constant, including the mass ratio of PAN to NaOH of 1, the concentration of NaOH solution of 8%, the hydrolysis time of 3 h, and the hydrolysis temperature of 105 °C. The addition order of raw material solution had no significant impact on the formation of the HPAN-CTAC complex, as illustrated in Figure 3a–c. This phenomenon could be attributed to the significant disparity in conductivity values between HPAN and CTAC. Regardless of the order in which they were added, the system’s conductivity values were consistently dominated by HPAN.

### 3.3. Effects of Alkyl Chain Length on PESC System

In addition to electrostatic interactions, hydrophobic interactions also play a significant role in the interaction between polyelectrolytes and surfactants [5,32]. For surfactant homologues, the hydrophobicity of the surfactant increases with the lengthening of the alkyl chain [33]. 

**Figure 4:**
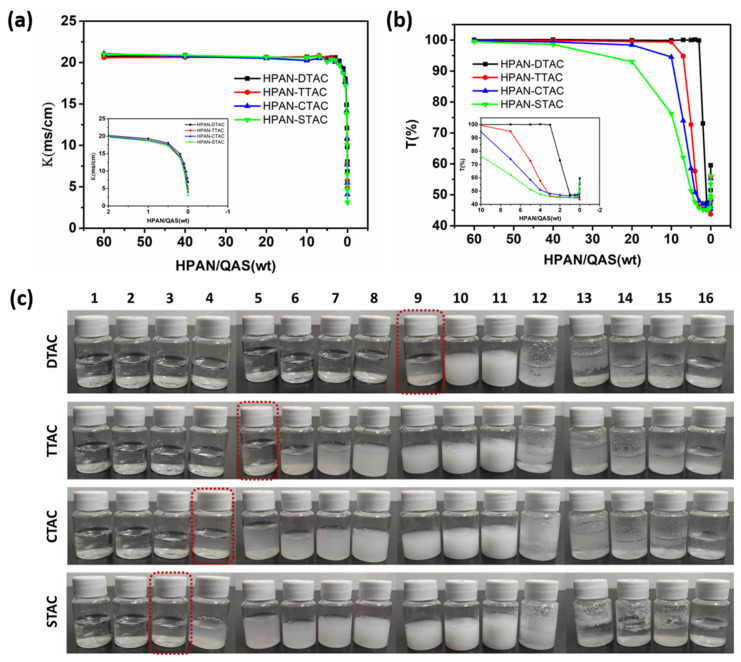
(**a**) Conductivity and (**b**) T% of HPAN-QAS solution with different alkyl chain lengths. (**c**) Photographs of the transformation of HPAN-QAS solution (the point at which the solution system begins to exhibit turbidity is indicated by the dotted red box).

To investigate the impact of alkyl chain lengths in surfactants on a complex formation, we selected DTAC, TTAC, CTAC, and STAC as the surfactants with varying alkyl chain lengths (C12, C14, C16, and C18, respectively). The hydrolysis of PAN was carried out under the following conditions: NaOH-to-PAN mass ratio of 1:1, a NaOH solution concentration of 8%, a hydrolysis time of 3 h, and a hydrolysis temperature of 105 °C. As depicted in Figure 4a, the addition of four different surfactants did not significantly affect the conductivity of the HPAN-QAS composite system. This might be attributed to the minimal discrepancy in conductivity values among DTAC (6862 us/cm), TTAC (4770 us/cm), CTAC (3974 us/cm), and STAC (3178 us/cm) solutions (Figure S2). 

However, the influence of the alkyl chain length on the transmittance of the HPAN-QAS composite system was evident, as shown in Figure 4b,c. The order of mixed aggregates for the four surfactants with polyelectrolyte is as follows: STAC > CTAC > TTAC > DTAC. The formation of the HPAN-QAS complex was primarily governed by electrostatic interactions between HPAN and QAS. The hydrophobic effect becomes stronger as the alkyl chain lengthens, leading to accelerated complex formation in the HPAN-STAC system and a reduction in solution transmittance.

Additionally, surfactant self-assembly is also a contributing factor. Surfactant self-assembly is governed by a balance between hydrophobic attractions and electrostatic repulsions [34]. The longer the alkyl chain, the greater the propensity for self-assembly in hydrophobic interactions, as typically observed through the critical micelle concentration (CMC) [35]. The CMC of the four surfactants can be observed in Figure S1, DTAC > TTAC > CTAC > STAC. When dissolved in water, STAC exhibited a higher tendency to form micelles and react with HPAN more readily, resulting in the accelerated formation of the HPAN-STAC complex.

In conclusion, the hydrophobicity of a surfactant is influenced by its alkyl chain. On one hand, enhanced hydrophobicity facilitates the interaction between the surfactant and polyelectrolyte. On the other hand, surfactants with high hydrophobicity readily undergo self-assembly to form micelles that can rapidly aggregate with polyelectrolytes.

### 3.4. Characterization of Solid PESCs

**Figure 5:**
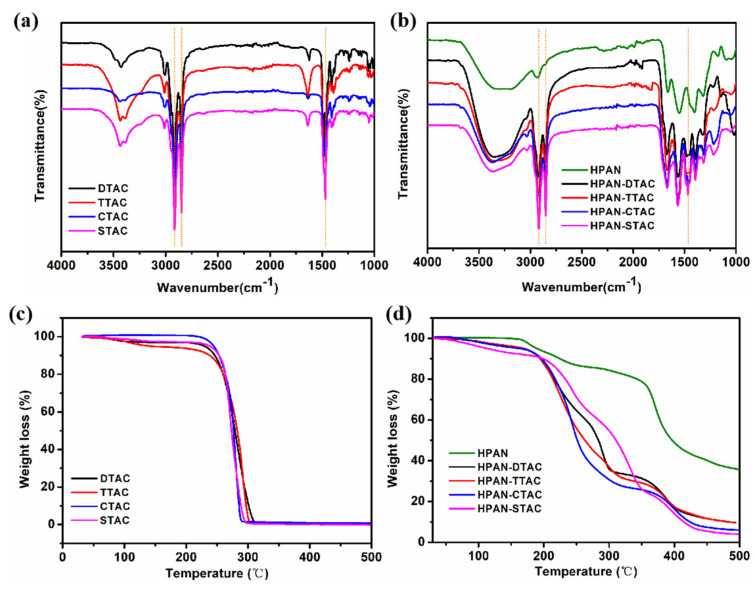
(**a**,**b**) FTIR spectra and (**c**,**d**) TGA curves of related samples.

The above results have demonstrated that the formation of PESCs was greatly influenced by the properties of polyelectrolytes and surfactants. In the next step, the PESC solid was separated through filtration, followed by vacuum drying at 60 °C in a drying oven, and finally, it was stored in a drying dish for future property analysis. The infrared spectra of PESCs are shown in Figure 5a,b. Characteristic absorption bands for HPAN were observed as follows: a band at 3350 cm^−1^, which could be attributed to the stretching vibration of -OH; two bands at about 1666 cm^−1^ and 1563 cm^−1^ that might be ascribed to the stretching vibration of C=O in the amide band; and the asymmetric stretching vibration of C=O in the carboxylate group, respectively. The obtained results suggest that the nitrile groups (-C≡N) undergo transformation into amide (-CONH_2_) and carboxylic groups (-COOH), as documented in the literature [29,36,37]. The QAS samples (DTAC, TTAC, CTAC, and STAC) exhibited typical bands, as shown in Figure 5a, which displayed two prominent absorption peaks at 2915 and 2850 cm^−1^ corresponding to the stretching vibrations of CH_2_ groups. Additionally, the intensity of those two peaks was significantly higher in HPAN-QAS, qualitatively indicating a combination of HPAN and QAS. The band at 1472 cm^−1^, meanwhile, was attributed to the C-N stretching of QAS, which was also evident in the HPAN-QAS spectrum, thereby confirming the successful immobilization of QAS within HPAN.

The thermal stability of PESCs has been evaluated by TGA. As shown in Figure 5c,d, the first stage of weight loss for all the samples at about 160 °C was associated with the bound water molecules. The significant loss of all QAS primarily occurred between 220 °C and approximately 300 °C, which could be attributed to the thermal decomposition of QAS. The rapid degradation of HPAN-QAs primarily occurs within the temperature range of 160 to 350 °C, attributed to both the thermal decomposition of QAS and the dehydration reaction of the residual -COOH in HPAN following self-assembly with QAS [29,38]. Residual weight above 350 °C was from the breakdown of the polymer structure and their degradation. The residual carbon rate serves as an intuitive indicator of the variations in thermal stability exhibited by the samples [39]. The residual carbon rate decreased from 35.78% to below 10% following the combination with QAS, indicating a reduction in thermal stability while successfully incorporating QAS.

### 3.5. Characterization of PESCs and PESC/PCL NFs

**Figure 6:**
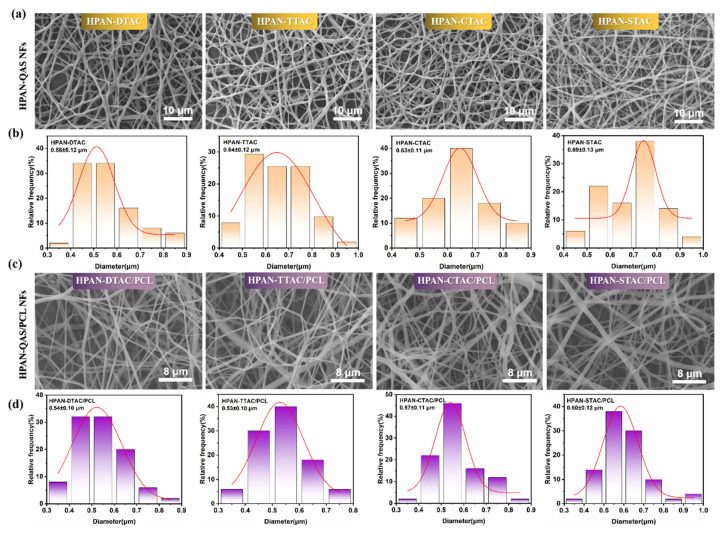
SEM images (**a**) and diameter distributions (**b**) of HPAN-QAS nanofibrous membranes; SEM images (**c**) and diameter distributions (**d**) of HPAN-QAS/PCL nanofibrous membranes.

Electrospinning was performed using the traditional single-nozzle spinneret setup. Continuous, cylindrical PESC fibers were successfully obtained, as shown in Figure 6a. The surface of all as-spun PESC fibers was smooth and without a beaded structure. Figure 6b shows the diameter distributions of HPAN-QASs nanofibers. The observed diameter ranged from 0.56 to 0.69 μm, with relatively small differences in size. After PAN hydrolysis, numerous hydrophilic groups were generated, leading to an increase in the hygroscopicity of PESCs. Figure S3 clearly demonstrates the evident adhesion between nanofibers of PESCs after water absorption, rendering the fiber morphology nearly imperceptible. To improve the structural stability and enhance the application potential of PESC fibrous membranes, polycaprolactone (PCL) was selected as an additional component for blending. The electrospun polymer PCL, widely employed in various biological and medical fields, demonstrates extensive applications due to its inherent hydrophobic properties, exceptional biocompatibility, and favorable mechanical characteristics [40]. The representative SEM images of the relevant nanofibrous membranes are shown in Figure 6c, which revealed smoother, non-beaded, and randomly oriented three-dimensional non-woven morphology for PESCs/PCL fibers. The swelling properties of fibers were improved after the mixture of PCL. The fiber unevenness, however, was more pronounced, potentially attributed to phase separation resulting from the asymmetric wettability of the two materials during the electrospinning process. The diameter of PESCs/PCL nanofibers exhibited a slight reduction compared to that of PESC nanofibers, as shown in Figure 6d.

### 3.6. Mechanical Analysis

**Figure 7:**
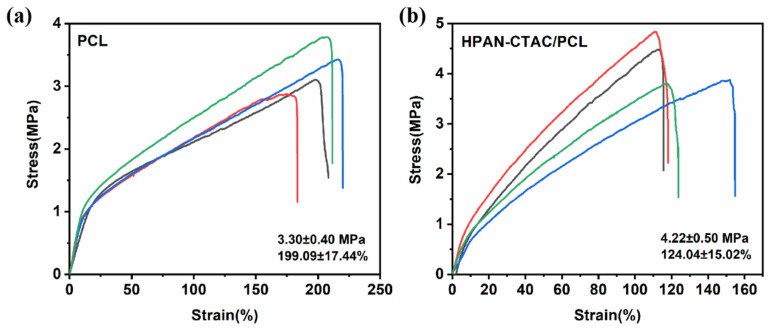
Mechanical properties of PCL (**a**) and HPAN-CTAC/PCL (**b**) nanofibrous membranes.

The consideration of mechanical properties is crucial in practical applications. Figure 7 illustrates the tensile curves of the HPAN-CTAC/PCL and PCL nanofibrous membranes, and four experiments were performed for each sample. It is worth noting that the pure PESC nanofiber membranes exhibited adhesion during the strength test due to their exceptional moisture absorption capability, which hindered the acquisition of accurate experimental results. The tensile strength of pure PCL was measured to be 3.3 MPa, as depicted in Figure 7a, while the elongation at break reached an impressive value of 199.09%, indicating excellent flexibility. For PESC/PCL nanofibrous membranes, as shown in Figure 7b, the tensile strength was increased to 4.22 MPa, and the strain was decreased to about 124%. Possibly due to the modification of quaternary ammonium salt, the movement and arrangement of polymer molecular chains during the stretching process were hindered, thereby enhancing the tensile strength and stiffness of the nanofibrous membranes. The application of fibrous membranes necessitates their possession of favorable tensile strength and elongation at break, such as for use in dressings and filtration materials. Furthermore, the obtained tensile properties of PESC/PCL membranes exhibited similarities to other reported values for antibacterial nanofiber materials based on PAN or PCL, suggesting their potential in practical applications [41,42,43]. 

### 3.7. Antibacterial Activity of PESC/PCL NFs

**Figure 8:**
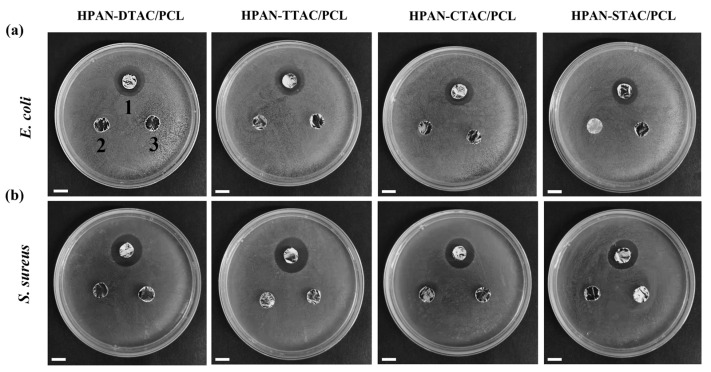
Inhibition zone of HPAN-QAS/PCL (1), PCL (2), and control (3) nanofibrous membranes toward *E. coli* (**a**) and *S. aureus* (**b**). The scale bar is 1 cm.

The antibacterial properties of PESCs/PCL fibers were studied on *E. coli* and *S. aureus* using the inhibition and shake flask method. As shown in Figure 8, pure PCL and control samples did not exhibit any inhibition activities against two kinds of bacteria. In contrast, the apparent inhibition zone could be observed for PESCs/PCL membranes, indicating effective antibacterial activities against both *E. coli* and *S. aureus*. We concluded that the ionic interaction between HPAN and QAS results in the diffusion of QAS, which leads to the inhibition zones. Furthermore, the negative bacteria membranes will be electrostatically attracted by the positive surface of PESCs/PCL fibers. Then the quaternary ammonium groups of QAS will disrupt the bacteria membranes, causing their death.

**Figure 9:**
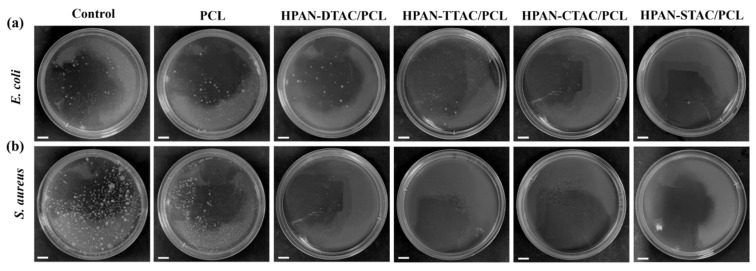
Photographs of agar plates of HPAN-QAS/PCL, PCL, and control nanofibrous membranes toward *E. coli* (**a**) and *S. aureus* (**b**). The scale bar is 1 cm.

The plate method was further adopted to evaluate the antibacterial properties of the PESCs/PCL fibers. As shown in Figure 9, the PCL nanofibrous membranes exhibited no inhibitory effect against *E. coli* and *S. aureus*. The PESC/PCL composite nanofibrous membranes, however, exhibited remarkable bactericidal effects against both *E. coli* and *S. aureus*, demonstrating exceptional antibacterial efficiency. The finding suggested that the blended PESC/PCL fiber materials still maintain excellent antibacterial properties. During the spinning process, a portion of PESC polymers tends to migrate towards the surface of composite nanofibers, facilitating sustained release or contact-based bactericidal activity by the antibacterial components inherent in PESC materials. The antibacterial activity is comparable to that achieved by Gliscinska et al., who also obtained excellent antibacterial effects through the physical doping of PAN solutions with quaternary ammonium salts to prepare nanofibers [44]. The materials prepared in our study exploit the electrostatic and hydrophobic interactions between polyelectrolytes and quaternary ammonium salts, which may offer certain advantages in terms of antimicrobial durability.

## 4. Conclusions

In conclusion, the interaction between hydrolyzed PAN and cationic surfactants was influenced by the concentration of the NaOH solution, the mass ratio of PAN-NaOH, the addition sequence, and the alkyl chain length. By means of electrospinning, PESCs and PESCs/PCL nanofibrous membranes with smooth and continuous surface morphologies were successfully obtained. Moreover, an antibacterial activity assessment demonstrated that the PESCs/PCL nanofibrous membranes exhibited excellent antibacterial activities against both *E. coli* and *S. aureus*, making them suitable for various applications such as wound dressing, water filtration, and protective equipment.

## Data Availability

Data presented in this study are available on request from the corresponding author.

## Supplementary Materials

The following supporting information can be downloaded at: https://www.mdpi.com/article/10.3390/polym16030414/s1, Figure S1: Molar conductivity - square root of concentration relationship curve; Figure S2: Conductivity of DTAC, TTAC, CTAC, and STAC; Figure S3: SEM image of the PESCs nanofibrous membrane after absorbing water; Table S1: Summary of the experimental description for additions of HPAN and QAS.

## References

[1] Miyake M. (2017). Recent progress of the characterization of oppositely charged polymer/surfactant complex in dilution deposition system. Adv. Colloid Interface Sci..

[2] Langevin D. (2009). Complexation of oppositely charged polyelectrolytes and surfactants in aqueous solutions. A review. Adv. Colloid Interface Sci..

[3] Madinya J.J., Tjo H., Meng X., Ramírez Marrero I.A., Sing C.E., Perry S.L. (2023). Surface Charge Density and Steric Repulsion in Polyelectrolyte–Surfactant Coacervation. Macromolecules.

[4] Yin C., Lin Z., Jiang X., Martin N., Tian L. (2023). Engineering the Coacervate Microdroplet Interface via Polyelectrolyte and Surfactant Complexation. ACS Appl. Mater. Interfaces.

[5] Khan N., Brettmann B. (2019). Intermolecular Interactions in Polyelectrolyte and Surfactant Complexes in Solution. Polymers.

[6] Llamas S., Guzmán E., Ortega F., Baghdadli N., Cazeneuve C., Rubio R.G., Luengo G.S. (2015). Adsorption of polyelectrolytes and polyelectrolytes-surfactant mixtures at surfaces: A physico-chemical approach to a cosmetic challenge. Adv. Colloid Interface Sci..

[7] Benhur A.M., Diaz J., Amin S. (2021). Impact of polyelectrolyte-surfactant interactions on the rheology and wet lubrication performance of conditioning shampoo. Int. J. Cosmet. Sci..

[8] Schmitt C., Turgeon S.L. (2011). Protein/polysaccharide complexes and coacervates in food systems. Adv. Colloid Interface Sci..

[9] Sharipova A., Aidarova S., Grigoriev D., Mutalieva B., Madibekova G., Tleuova A., Miller R. (2016). Polymer–surfactant complexes for microencapsulation of vitamin E and its release. Colloids Surf. B Biointerfaces.

[10] de Silva U.K., Brown J.L., Lapitsky Y. (2018). Poly(allylamine)/tripolyphosphate coacervates enable high loading and multiple-month release of weakly amphiphilic anionic drugs: An in vitro study with ibuprofen. RSC Adv..

[11] Mirtič J., Kogej K., Baumgartner S., Smistad G., Kristl J., Hiorth M. (2016). Development of Cetylpyridinium-Alginate Nanoparticles: A Binding and Formulation Study. Int. J. Pharm..

[12] Homaeigohar S., Boccaccini A.R. (2020). Antibacterial biohybrid nanofibers for wound dressings. Acta Biomater..

[13] Zhao K., Kang S.-X., Yang Y.-Y., Yu D.-G. (2021). Electrospun Functional Nanofiber Membrane for Antibiotic Removal in Water: Review. Polymers.

[14] Lu X., Wang C., Wei Y. (2009). One-dimensional composite nanomaterials: Synthesis by electrospinning and their applications. Small.

[15] Donner R., Potirakis S., Balasis G., Eftaxias K., Kurths J. (2023). Electrospun fiber-based strategies for controlling early innate immune cell responses: Towards immunomodulatory mesh designs that facilitate robust tissue repair. Phys. Chem. Earth.

[16] Bassalah M.E., Cerdà J.J., Sintes T., Aschi A., Othman T. (2017). Complex between cationic like-charged polyelectrolytes/surfactants systems. Eur. Polym. J..

[17] Durmaz E.N., Sahin S., Virga E., de Beer S., de Smet L.C.P.M., de Vos W.M. (2021). Polyelectrolytes as Building Blocks for Next-Generation Membranes with Advanced Functionalities. ACS Appl. Polym. Mater..

[18] Josef E., Guterman R. (2019). Designing Solutions for Electrospinning of Poly(ionic liquid)s. Macromolecules.

[19] Meng X., Perry S.L., Schiffman J.D. (2017). Complex Coacervation: Chemically Stable Fibers Electrospun from Aqueous Polyelectrolyte Solutions. ACS Macro Lett..

[20] Meng X., Schiffman J.D., Perry S.L. (2018). Electrospinning Cargo-Containing Polyelectrolyte Complex Fibers: Correlating Molecular Interactions to Complex Coacervate Phase Behavior and Fiber Formation. Macromolecules.

[21] Kriegel C., Kit K., McClements D., Weiss J. (2009). Electrospinning of chitosan–poly(ethylene oxide) blend nanofibers in the presence of micellar surfactant solutions. Polymer.

[22] Kenawy E.R., Bowlin G.L., Mansfield K., Layman J., Simpson D.G., Sanders E.H., Wnek G.E. (2002). Release of tetracycline hydrochloride from electrospun poly(ethylene-co-vinylacetate), poly(lactic acid), and a blend. J. Control. Release.

[23] Poudel M.B., Kim A.A. (2023). Silver nanoparticles decorated TiO_2_ nanoflakes for antibacterial properties. Inorg. Chem. Commun..

[24] Qiu Q., Chen S., Li Y., Yang Y., Zhang H., Quan Z., Qin X., Wang R., Yu J. (2020). Functional nanofibers embedded into textiles for durable antibacterial properties. Chem. Eng. J..

[25] Kono H., Sogame Y., Purevdorj U.-E., Ogata M., Tajima K. (2023). Bacterial cellulose nanofibers modified with quaternary ammonium salts for antimicrobial applications. ACS Appl. Nano Mater..

[26] Shi R., Geng H., Gong M., Ye J., Wu C., Hu X., Zhang L. (2018). Long-acting and broad-spectrum antimicrobial electrospun poly (ε-caprolactone)/gelatin micro/nanofibers for wound dressing. J. Colloid Interface Sci..

[27] Chen T., Zhao L., Wang Z., Zhao J., Li Y., Long H., Yu D., Wu X., Yang H. (2020). Hierarchical Surface Inspired by Geminized Cationic Amphiphilic Polymer Brushes for Super-Antibacterial and Self-Cleaning Properties. Biomacromolecules.

[28] Qiu Q., Wu J., Quan Z., Zhang H., Qin X., Wang R., Yu J. (2019). Electrospun nanofibers of polyelectrolyte–surfactant complexes for antibacterial wound dressing application. Soft Matter.

[29] Jin S.Y., Kim M.H., Jeong Y.G., Yoon Y.I., Park W.H. (2017). Effect of alkaline hydrolysis on cyclization reaction of PAN nanofibers. Mater. Des..

[30] Lai C.-L., Chao W.-C., Hung W.-S., An Q., De Guzman M., Hu C.-C., Lee K.-R. (2015). Physicochemical effects of hydrolyzed asymmetric polyacrylonitrile membrane microstructure on dehydrating butanol. J. Membr. Sci..

[31] Goddard E.D. (1986). Polymer-surfactant interaction: Part II: Polymer and surfactant of opposite charge. Interactions of Surfactants with Polymers and Proteins.

[32] Gradzielski M., Gradzielski M. (2022). Polyelectrolyte–surfactant complexes as a formulation tool for drug delivery. Langmuir.

[33] Shaw B.F., Schneider G.F., Whitesides G.M. (2012). Effect of surfactant hydrophobicity on the pathway for unfolding of ubiquitin. J. Am. Chem. Soc..

[34] Gonçalves R.A., Holmberg K., Lindman B. (2023). Cationic surfactants: A review. J. Mol. Liq..

[35] Kronberg B. (2016). The hydrophobic effect. Curr. Opin. Colloid Interface Sci..

[36] Fang J., Liu G., Chen C., Lin C., Zhang B., Jin H., Chen Y., Lu J., Zhu L. (2021). Intrinsically antibacterial thin film composite membranes with supramolecularly assembled lysozyme nanofilm as selective layer for molecular separation. Sep. Purif. Technol..

[37] Xu D., Pan G., Ge Y., Yang X. (2023). Preparation of a low-protein-fouling and high-protein-retention membrane via novel pre-hydrolysis treatment of polyacrylonitrile (PAN). Membranes.

[38] Kennedy J.P., Fontana C.M. (1959). The thermal degradation of polyacrylonitrile. J. Polym. Sci..

[39] Xu C.-A., Qu Z., Meng H., Chen B., Wu X., Cui X., Wang K., Wu K., Shi J., Lu M. (2021). Effect of polydopamine-modified multi-walled carbon nanotubes on the thermal stability and conductivity of UV-curable polyurethane/polysiloxane pressure-sensitive adhesives. Polymer.

[40] Goudarzi Z.M., Soleimani M., Ghasemi-Mobarakeh L., Sajkiewicz P., Sharifianjazi F., Esmaeilkhanian A., Khaksar S. (2022). Control of drug release from cotton fabric by nanofibrous mat. Int. J. Biol. Macromol..

[41] Ullah S., Hashmi M., Kharaghani D., Khan M.Q., Saito Y., Yamamoto T., Lee J., Kim I.S. (2019). Antibacterial properties of in situ and surface functionalized impregnation of silver sulfadiazine in polyacrylonitrile nanofiber mats. Int. J. Nanomed..

[42] Augustine R., Dominic E.A., Reju I., Kaimal B., Kalarikkal N., Thomas S. (2015). Electrospun poly(ε-caprolactone)-based skin substitutes: *In vivo* evaluation of wound healing and the mechanism of cell proliferation. J. Biomed. Mater. Res. Part B Appl. Biomater..

[43] Aksoy O.E., Ates B., Cerkez I. (2017). Antibacterial polyacrylonitrile nanofibers produced by alkaline hydrolysis and chlorination. J. Mater. Sci..

[44] Gliścińska E., Gutarowska B., Brycki B., Krucińska I. (2013). Electrospun polyacrylonitrile nanofibers modified by quaternary ammonium salts. J. Appl. Polym. Sci..

