# Supplementary material for: Polyelectrolyte–Surfactant Complex Nanofibrous Membranes for Antibacterial Applications
Source: Polymers (Basel). 2024 Feb 1;16(3):414. doi: 10.3390/polym16030414 (PMC10857514; doi:10.3390/polym16030414)
Supplement: Supplementary file 1 [file polymers-16-00414-s001.zip › polymers-2826033-supplementary.pdf]

Polyelectrolyte–Surfactant Complex Nanofibrous Membranes for Antibacterial Applications

Qiaohua Qiu *, Zhengkai Wang and Liying Lan

College of Textile Science and Engineering, Zhejiang Sci-Tech University, Hangzhou 310018, China; wangzhengkaiii@163.com (Z.W.); lanying2024@163.com (L.L.)

Table S1. Summary of the experimental description for additions of HPAN and QAS.

	HPAN/QAS mass ratio (wt/wt)	HPAN/QAS volume ratio (v/v)	HPAN (mL)	QAS (mL)
1	60	300	12	0.04
2	40	200	12	0.06
3	20	100	10	0.1
4	10	50	10	0.2
5	7	35	14	0.4
6	5	25	10	0.4
7	4	20	12	0.6
8	3	15	12	0.8
9	2	10	10	1
10	1	5	10	2
11	0.5	2.5	10	4
12	0.2	1	5	5
13	0.1	0.5	5	10
14	0.05	0.25	2	8
15	0.02	0.1	1	10
16	0.002	0.01	0.1	10

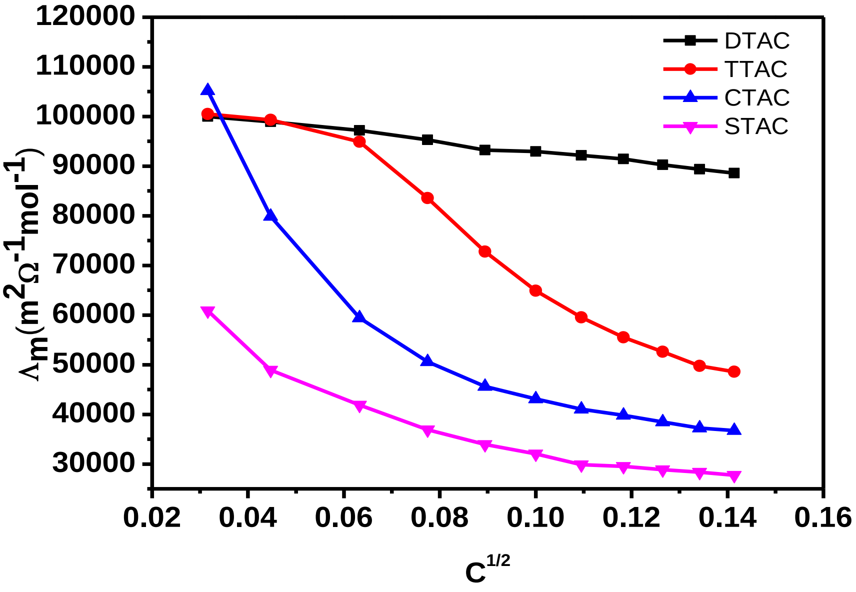

Figure S1. Molar conductivity - square root of concentration relationship curve.

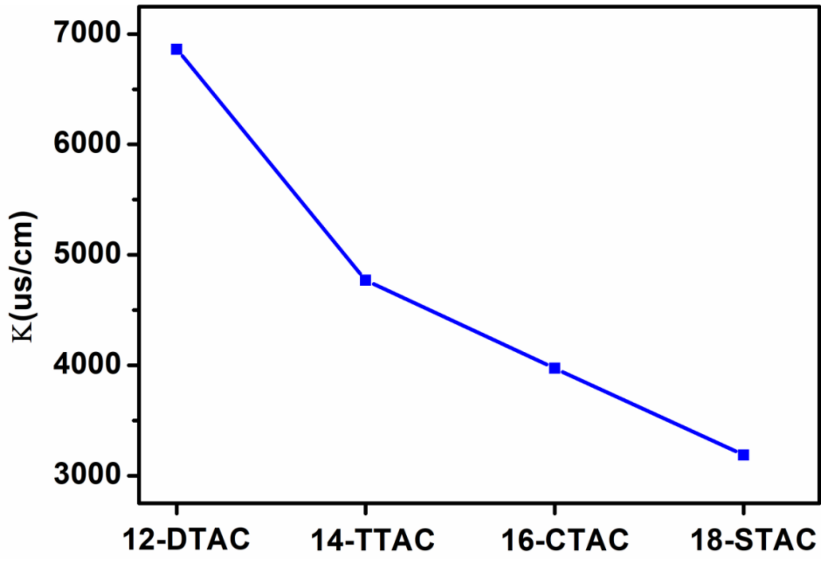

Figure S2. Conductivity of DTAC, TTAC, CTAC, and STAC.

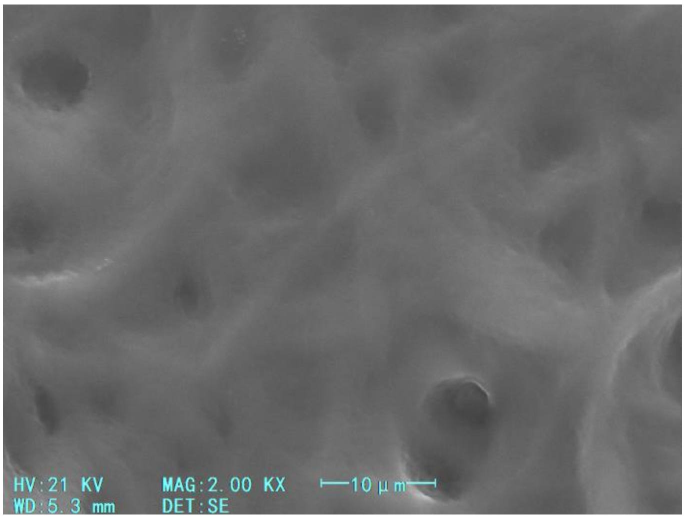

Figure S3. SEM image of the PESCs nanofibrous membrane after absorbing water.
